# Supplementary material for: Ecological, Genetic, and Phylogenetic Aspects of YFV 2017–2019 Spread in Rio de Janeiro State
Source: Viruses. 2023 Feb 4;15(2):437. doi: 10.3390/v15020437 (PMC9961572; doi:10.3390/v15020437)
Supplement: Supplementary file 1 [file viruses-15-00437-s001.zip › viruses-2106744-supplementary.pdf]

Clade	GenBank	Host	Date	Polyprotein position																								
				C				prM		E	NS1						NS2A	NS2B	NS3			NS4A	NS4B	NS5				
				79	82	103	108	131	254	526	821	826	829	978	993	1086	1313	1417	1503	1744	1831	2176	2502	2644	2764	2897	3128	3151
	MK333804	NHP	17 August 2015	V	R	Q	I	L	A	T	A	A	E	F	T	I	S	E	Y	E	E	I	K	I	I	N	I	T
YFV RJ I	MN643090	NHP	04 February 2018	.	.	.	.	.	.	.	.	.	.	.	.	.	.	.	.	.	.	.	.	.	.	.	.	.
YFV RJ I	MN643092	NHP	07 February 2018	.	.	.	.	.	.	A	.	.	.	.	.	.	.	.	.	.	.	.	.	.	.	.	.	.
YFV RJ I	MN643091	NHP	07 February 2018	.	.	.	.	.	.	.	.	.	.	.	.	.	.	.	.	.	.	.	.	.	.	.	.	.
YFV RJ I	MK333805	M	07 February 2018	.	.	.	.	.	.	.	.	.	.	.	.	.	.	.	.	.	.	.	.	.	.	.	.	.
YFV RJ I	MN506266	M	09 January 2018	.	.	.	.	.	.	.	.	.	.	.	.	.	.	.	.	.	.	.	.	.	.	.	.	.
YFV RJ I	MN643085	H	11 February 2018	.	.	.	.	.	.	.	.	.	.	.	.	.	.	.	.	.	.	.	.	.	.	.	.	.
YFV RJ I	MN643086	H	14 February 2018	.	.	.	.	.	.	.	.	.	.	.	.	.	.	.	.	.	.	.	.	.	V	.	.	.
YFV RJ I	MN643087	H	13 February 2018	.	.	.	.	.	.	.	.	.	.	.	.	.	.	.	.	.	.	.	.	.	.	.	.	.
YFV RJ I	MN643089	NHP	19 October 2017	.	.	.	.	.	.	.	.	.	.	.	.	.	.	.	.	.	.	.	.	.	.	.	.	.
YFV RJ I	MF538786	NHP	05 June 2017	.	.	.	.	.	.	.	.	.	.	.	.	.	.	.	.	.	.	.	.	.	.	.	.	.
YFV RJ I	MN506274	M	06 May 2017	.	.	.	.	.	.	.	.	.	.	L	.	.	.	.	.	.	.	.	.	.	.	.	.	.
YFV RJ I	MN506269	M	06 May 2017	.	.	.	.	.	.	.	.	.	.	L	.	.	.	.	.	.	.	.	.	.	.	.	.	.
YFV RJ I	MN506270	M	05 May 2017	.	.	.	.	.	.	.	.	.	.	L	.	.	.	.	.	.	.	.	.	.	.	.	.	.
YFV RJ I	MN506272	M	06 May 2017	.	.	.	.	.	.	.	.	.	.	L	.	.	.	.	.	.	.	.	.	.	.	.	.	.
YFV RJ I	MN506283	M	05 May 2017	.	.	.	.	.	.	.	.	.	.	.	.	.	.	.	.	.	.	.	.	.	.	.	.	.
YFV RJ I	MN506273	M	05 May 2017	.	.	.	.	.	.	.	.	.	.	.	.	.	.	.	.	.	.	.	.	.	.	.	.	.
YFV RJ I	MN506282	M	08 May 2017	.	.	.	.	.	.	.	.	.	.	.	.	.	.	.	.	.	.	.	.	.	.	.	.	.
YFV RJ I	MN506281	M	26 April 2017	.	.	.	.	.	.	.	S	.	.	.	.	.	.	.	.	.	.	.	.	.	.	.	.	.
YFV RJ I	MN643088	NHP	19 April 2017	.	.	.	.	.	.	.	.	.	.	.	.	.	.	.	.	.	.	.	.	.	.	.	.	.
YFV RJ I	MN506268	M	11 May 2017	.	.	.	.	.	.	.	.	.	.	.	.	.	.	.	.	.	.	.	.	.	.	.	.	.
YFV RJ I	MN506279	M	04 May 2017	.	.	.	.	.	.	.	.	.	.	.	.	.	.	.	.	.	.	.	.	.	.	.	.	.
YFV RJ I	MN506271	M	05 May 2017	.	.	.	.	.	.	.	.	.	.	.	.	.	.	G	.	.	.	.	.	.	.	.	.	.
YFV RJ I	MN506280	M	07 May 2017	.	.	.	.	.	.	.	.	.	.	.	.	.	.	.	.	.	.	.	.	.	.	.	.	.
YFV RJ I	MN506267	M	08 May 2017	.	.	.	.	.	.	.	.	.	.	.	.	.	.	.	.	.	.	.	.	T	.	.	.	.
YFV RJ I	MN506277	M	05 May 2017	.	.	.	.	.	.	.	.	.	.	.	I	.	.	.	.	.	.	.	.	.	.	.	.	.
YFV RJ I	MK533792	NHP	10 January 2019	.	.	R	.	.	.	.	.	.	D	.	.	.	.	.	.	G	.	.	.	.	.	K	M	I
YFV RJ I	MK882603	H	24 April 2018	.	.	.	.	.	.	.	.	.	.	.	.	.	.	.	.	G	.	.	.	.	.	.	M	.
YFV RJ I	MN506275	M	05 May 2017	.	.	.	.	.	.	.	.	.	.	.	.	.	.	.	.	.	.	.	.	.	.	.	.	.
YFV RJ I	MF423375	NHP	04 April 2017	.	.	.	.	.	.	.	.	.	.	.	.	.	.	.	.	.	.	.	.	.	.	.	.	.
YFV RJ I	MF423376	NHP	13 April 2017	.	.	.	.	.	.	.	.	.	.	.	.	.	.	.	.	.	.	.	.	.	.	.	.	.
YFV RJ I	MF434851	H	25 April 2017	.	.	.	.	.	.	.	.	S	.	.	.	.	.	.	.	.	.	.	.	.	.	.	.	.
YFV RJ I	MF538783	H	18 March 2017	.	.	.	.	.	.	.	.	.	.	.	.	.	.	.	.	.	.	V	.	.	.	.	.	.
YFV RJ I	MN643081	H	19 February 2018	.	.	.	.	.	.	.	.	.	.	.	.	.	.	.	.	.	.	.	.	.	.	.	.	.
YFV RJ I	MN643082	H	16 February 2018	.	.	.	.	S	.	.	.	.	.	.	.	.	.	.	.	.	.	.	.	.	.	.	.	.
YFV RJ I	MN506276	M	05 May 2017	.	.	.	.	.	.	.	.	.	.	.	.	.	.	.	.	.	.	.	.	.	.	.	.	.
YFV RJ I	MN506278	M	07 May 2017	.	.	.	.	.	.	.	.	.	.	.	.	.	.	.	.	.	.	.	.	.	.	.	.	.
YFV RJ II	MN506288	M	24 January 2018	.	.	.	.	.	.	.	.	.	.	.	.	V	.	.	.	.	.	V	.	.	.	.	.	.
YFV RJ II	MN506287	M	24 January 2018	.	.	.	.	.	.	.	.	.	.	.	.	V	.	.	.	.	.	V	.	.	.	.	.	.
YFV RJ II	MN506289	M	26 January 2018	.	.	.	.	.	.	.	.	.	.	.	.	V	.	.	.	.	.	V	.	.	.	.	.	.
YFV RJ II	MN506291	M	26 January 2018	.	.	.	.	.	.	.	.	.	.	.	.	V	.	.	.	.	.	V	.	.	.	.	.	.
YFV RJ II	MN506286	M	24 January 2018	.	.	.	.	.	.	.	.	.	.	.	.	V	.	.	.	.	.	V	.	.	.	.	.	.
YFV RJ II	MN506285	M	24 January 2018	.	.	.	.	.	.	.	.	.	.	.	.	V	.	.	.	.	.	V	.	.	.	.	.	.
YFV RJ II	MN506284	M	18 January 2018	.	.	.	.	.	.	.	.	.	.	.	.	V	.	.	.	.	.	V	.	.	.	.	.	.
YFV RJ II	MK333809	M	19 January 2018	.	.	.	.	.	.	.	.	.	.	.	.	.	.	.	.	.	.	V	.	.	.	.	.	.
YFV RJ II	MN506290	M	18 January 2018	.	.	.	.	.	.	.	.	.	.	.	.	.	.	.	.	.	.	V	.	.	.	.	.	.
YFV RJ II	MN643077	H	16 January 2018	.	.	.	.	.	.	.	.	.	.	.	.	.	.	.	.	.	.	V	.	.	.	.	.	.
YFV RJ II	MN643078	H	25 January 2018	.	.	.	.	.	.	.	.	.	.	.	.	.	.	.	.	.	.	V	.	.	.	.	.	.
YFV RJ II	MN506265	M	19 December 2017	.	.	.	.	.	.	.	.	.	.	.	.	.	.	.	.	.	.	V	.	.	.	.	.	.
YFV RJ II	MN643080	H	17 February 2018	.	.	.	.	.	.	.	.	.	.	.	.	.	.	.	.	.	.	V	.	.	.	.	.	.
YFV RJ II	MN643079	H	26 January 2018	.	.	.	.	.	.	.	.	.	.	.	.	.	.	.	.	.	.	V	.	.	.	.	.	.
YFV RJ II	MF423378	NHP	19 April 2017	.	.	.	.	.	.	.	.	.	.	.	.	.	.	.	.	.	.	V	.	.	.	.	.	.
YFV RJ II	MF423377	NHP	19 April 2017	.	.	.	.	.	.	.	.	.	.	.	.	.	.	.	.	.	.	V	.	.	.	.	.	.
YFV RJ II	MF538785	NHP	21 April 2017	.	.	.	.	.	.	.	.	.	.	.	.	.	.	.	.	.	.	V	.	.	.	.	.	.
	MF538784	H	26 February 2017	.	.	.	.	.	.	.	.	.	.	.	.	.	.	.	.	.	.	.	.	.	.	.	.	.
	MN643084	H	25 February 2018	.	.	.	.	.	.	.	.	.	.	.	.	.	L	.	H	.	G	.	E	.	.	.	.	.
	MN643083	H	25 February 2018	.	.	.	.	.	.	.	.	.	.	.	.	.	.	.	.	.	G	.	E	.	.	.	.	.
	MF538782	H	16 March 2017	.	.	.	.	.	.	.	.	.	.	.	.	.	.	.	.	.	.	.	.	.	.	.	.	.
	MK882607	H	09 March 2017	.	.	.	.	.	.	.	.	.	.	.	.	.	.	.	.	.	.	.	.	.	.	.	.	.
	MG550109	H	18 April 2017	A	K	.	V	.	V	.	.	.	.	.	.	.	.	.	.	.	.	.	.	.	.	.	.	.

Figure S1. Amino acid polymorphisms observed in the YFV polyproteins sampled in Rio de Janeiro State. The RJ sequences were aligned with the ancestral sequence MK333804 (strain GO27), which circulated in Goiás State in 2015 (dark blue). The variant amino acid residues are colored in green. The YFV samples belong to the clade RJ I are highlighted in light blue, while those that pertain to the RJ II clade are in orange. A group of RJ YFV clustered in ES clade (not colored). The type of infected host corresponds to: (H) human case, (NHP) non-human primate, and (M) mosquito.

Table S1. New YFV genome sequenced from the state Rio de Janeiro state. All samples were originated from localities in the Southeast Atlantic primary river basin, Atlantic Forest biome, Southeast Brazil.

Conjugated River Basin (Tributary Basin)	County	Geographic Coordinates	Strain	Date	Host	GenBank
Macaé Cconjugated Basin (Macaé)	Macaé	22°11'21.6"S 41°59'48.5"W	RJ98	2017-04-19	*A.clamitans*	MN643088
	Macaé	22°18'31.55"S 42°0'1.74"W	MC941	2017-04-26	*Hg.janthinomys*	MN506281
(São João)	Maricá	22°55'19.41"S 42°42'26.54"W	MR1438	2017-05-04	*Hg.leucocelaenus*	MN506279
	Maricá	22°55'19.41"S 42°42'26.54"W	MR 976	2017-05-05	*Hg.janthinomys*	MN506283
	Maricá	22°55'19.41"S 42°42'26.54"W	MR1139	2017-05-05	*Hg.leucocelaenus*	MN506270
	Maricá	22°55'19.41"S 42°42'26.54"W	MR1140	2017-05-05	*Hg.leucocelaenus*	MN506271
	Maricá	22°55'19.41"S 42°42'26.54"W	MR1145	2017-05-05	*Hg.leucocelaenus*	MN506277
	Maricá	22°55'19.41"S 42°42'26.54"W	MR1152	2017-05-05	*Hg.leucocelaenus*	MN506275
	Maricá	22°55'19.41"S 42°42'26.54"W	MR1158	2017-05-05	*Hg.leucocelaenus*	MN506273
	Maricá	22°55'19.41"S 42°42'26.54"W	MR1160	2017-05-05	*Hg.leucocelaenus*	MN506276
	Maricá	22°55'19.41"S 42°42'26.54"W	MR1353	2017-05-06	*Ae.scapularis*	MN506272
	Maricá	22°55'19.41"S 42°42'26.54"W	MR1356	2017-05-06	*Ae.taeniorhynchus*	MN506274
	Maricá	22°55'19.41"S 42°42'26.54"W	MR1351	2017-05-06	*Hg.leucocelaenus*	MN506269
	Maricá	22°55'19.41"S 42°42'26.54"W	MR1358	2017-05-07	*Hg.leucocelaenus*	MN506278
	Maricá	22°55'19.41"S 42°42'26.54"W	MR1391	2017-05-07	*Hg.leucocelaenus*	MN506280
	Maricá	22°55'19.41"S 42°42'26.54"W	MR 960	2017-05-08	*Hg.janthinomys*	MN506282
	Maricá	22°55'19.41"S 42°42'26.54"W	MR1044	2017-05-08	*Hg.leucocelaenus*	MN506267
	Maricá	22°55'19.41"S 42°42'26.54"W	MR1112	2017-05-11	*Hg.leucocelaenus*	MN506268
(Guandu)	Miguel Pereira	22°29'12.05"S 43°18'31.2"W	RJ123	2017-10-19	*A.clamitans*	MN643089
	Nova Iguaçu	22°35'0.00"S 43°24'33.00"W	NI3104	2018-01-09	*Hg.janthinomys*	MN506266
(Ilha Grande Bay)	Angra dos Reis - IG	23°08'19.8"S 44°18'47.9"W	RJ136	2018-02-04	*A. clamitans*	MN643090
	Angra dos Reis - IG	23°11'0.83"S 44°11'56.3"W	RJ138	2018-02-07	*A. clamitans*	MN643091
	Angra dos Reis - IG	23°11'0.83"S 44°11'56.3"W	RJ139	2018-02-07	*A. clamitans*	MN643092
	Angra dos Reis - IG	23°09'42.0"S 44°20'52.0"W	H326	2018-02-11	*H.sapiens*	MN643085
	Angra dos Reis - IG	23°09'42.0"S 44°20'52.0"W	H333	2018-02-13	*H.sapiens*	MN643087
	Angra dos Reis -CO	22°52'37.3"S 44°17'04.7"W	H327	2018-02-14	*H.sapiens*	MN643086
Paraíba do Sul Conjugated Basin (Paraíba do Sul)	Teresópolis	22°23'17.06"S 42°57'5.47"W	TR2807	2017-12-19	*Hg.janthinomys*	MN506265
	Teresópolis	22°23'53.03"S 42°56'49.6"W	H295	2018-01-16	*H.sapiens*	MN643077
	Teresópolis	22°15'17.6"S 42°45'07.9"W	H299	2018-01-25	*H.sapiens*	MN643078
	Teresópolis	22°10'19.02"S 42°51'22.2"W	H300	2018-01-26	*H.sapiens*	MN643079
	Teresópolis	22°26'20.0"S 42°56'17.4"W	H317	2018-02-16	*H.sapiens*	MN643082
	Teresópolis	22°16'23.08"S 42°47'58.2"W	H312	2018-02-17	*H.sapiens*	MN643080
	Teresópolis	22°26'15.9"S 42°56'19.2"W	H313	2018-02-19	*H.sapiens*	MN643081
	Teresópolis	22°26'17.0"S 42°56'51.3"W	H319	2018-02-25	*H.sapiens*	MN643083
	Teresópolis	22°26'17.0"S 42°56'51.3"W	H320	2018-02-25	*H.sapiens*	MN643084
	Valença	22°10'26.03"S 43°43'25.14"W	VL2909	2018-01-18	*Hg.janthinomys*	MN506290
	Valença	22°9'45.06"S 43°42'4.18"W	VL2967	2018-01-18	*Hg.janthinomys*	MN506284
	Valença	22°9'45.06"S 43°42'4.18"W	VL2953	2018-01-24	*Hg.janthinomys*	MN506285
	Valença	22°9'45.06"S 43°42'4.18"W	VL2964	2018-01-24	*Hg.janthinomys*	MN506288
	Valença	22°9'45.06"S 43°42'4.18"W	VL3005	2018-01-24	*Hg.janthinomys*	MN506286
	Valença	22°9'45.06"S 43°42'4.18"W	VL3006	2018-01-24	*Hg.janthinomys*	MN506287
	Valença	22°9'45.06"S 43°42'4.18"W	VL3012	2018-01-26	*Hg.janthinomys*	MN506289
	Valença	22°9'45.06"S 43°42'4.18"W	VL3016	2018-01-26	*Hg.janthinomys*	MN506291

CO: Continent; IG: Ilha Grande (island).

Table S2. YFV samples from Rio de Janeiro State (2017-2019).

Genbank	Strain	Host	Collection Date	Location (State)
MF538784	H196*	*Homo sapiens*	26/02/2017	Porciúncula
MK882607	RJ182**	*Alouatta sp*	09/03/2017	São Sebastião do Alto
MF538782	H190*	*Homo sapiens*	16/03/2017	São Fidélis
MF538783	H191*	*Homo sapiens*	18/03/2017	Casimiro de Abreu
MF423375	RJ87*	*Alouatta guariba*	04/04/2017	Macaé
MF423376	RJ94*	*Alouatta guariba*	13/04/2017	Macaé
MG550109	H189*	*Homo sapiens*	18/04/2017	Maricá
MF423377	RJ95*	*Alouatta guariba*	19/04/2017	Carmo
MF423378	RJ96*	*Alouatta guariba clamitans*	19/04/2017	Carmo
MF538785	RJ97*	*Callithrix sp.*	21/04/2017	Petrópolis
MF434851	H199*	*Homo sapiens*	25/04/2017	Casimiro de Abreu
MF538786	RJ104*	*Callithrix jacchus*	05/06/2017	Guapimirim
MK333809	VL2926***	*Hae. janthinomys*	19/01/2018	Valença
MK333805	IG3036***	*Sa.chloropterus*	07/02/2018	Ilha Grande - Angra dos Reis
MK882603	LABFLA10**	*Homo sapiens*	24/04/2018	Silva Jardim
MK533792	RJ155****	*Alouatta guariba*	10/01/2019	Casimiro de Abreu

*[20], **[46],***[19],****[15]. Strain= project identifier; Host=Host species; GenBank= GenBank accession number. Ilha Grande=IG (island).

Table S3. Comparison of continuous spatial models fit to the 2015-2018 Brazilian YFV dataset and estimate of $YFV_{2015\text{-}2018}$ lineage dispersal rate under the different models.

	**Homogeneous Brownian diffusion**	**HeterogeneousRRW Cauchy**	**HeterogeneousRRW Gamma**	**HeterogeneousRRW Lognormal**
PS - Log ML[a]	-18110	-18085	-18083	-18076/-18094/-18101
PS – Log BF[b]	34	9	7	Fittest model
SS - Log ML[a]	-18111	-18085	-18084	-18078/-18095/-18103
SS – Log BF[b]	33	7	6	Fittest model
Dispersal rate (km/day)[c]	0.3 (0.2- 0.5)	0.5 (0.4- 0.6)	0.5 (0.4- 0.6)	0.5 (0.4- 0.6)

[a] Log marginal likelihood (ML) estimates for the different continuous phylogeographic models obtained using the path- sampling (PS) and stepping-stone sampling (SS) methods. [b] The Log Bayes factor (BF) is the difference of the Log ML between of alternative (H1) and null (H0) models (H1/H0). Log BFs > 3 indicates that model H1 is more strongly supported by the data than model H0. [c] Posterior mean and 95% HPD (in parenthesis) estimates of the dispersal rate.
